# Increased Non-Homologous End Joining Makes DNA-PK a Promising Target for Therapeutic Intervention in Uveal Melanoma

**DOI:** 10.3390/cancers11091278

**Published:** 2019-08-30

**Authors:** Rachel E. Doherty, Helen E. Bryant, Manoj K. Valluru, Ian G. Rennie, Karen Sisley

**Affiliations:** 1 Department of Oncology and Metabolism, The Medical School, Beech Hill Road, Sheffield S10 2RX, UK; 2 Sheffield Ocular Oncology Service, Sheffield Teaching Hospitals, Royal Hallamshire Hospital, Glossop Road S10 2JF, UK

**Keywords:** uveal melanoma, DNA-PK, NHEJ, HR, PARP, chemo-sensitization

## Abstract

Uveal melanoma (UM) is the most common primary intraocular tumour in adults, with a mean survival of six months following metastasis. The survival rates have not improved in over 30 years. This study has shown that sister chromatid exchange (SCE) is low in UM which is likely due to a reduced expression of *FANCD2*. As *FANCD2* can function to suppress non-homologous end joining (NHEJ), this study therefore investigated NHEJ in UM. The activation of the catalytic subunit of the NHEJ pathway protein DNA-dependent protein kinase (DNA-PK) was measured by analysing the foci formation and the ligation efficiency by NHEJ determined using a plasmid-based end-joining assay. Using small-interfering RNA (siRNA) knock-down, and chemical inhibitors of DNA-PK, the survival of primary UM cultures and two cell lines were determined. To assess the homologous recombination capacity in response to the inhibition of DNA-PK, a SCE analysis was performed. In addition, to support the findings, the messenger RNA (mRNA) expression of genes associated with NHEJ was analysed using the Cancer Genome Atlas (TCGA)-UM RNAseq data (*n* = 79). The NHEJ activity and DNA-PKcs activation was upregulated in UM and the inhibition of DNA-PK selectively induced apoptosis and sensitized to ionising radiation and inter-strand cross-linking agents. The inhibition of the NHEJ protein DNA-PK is lethal to UM, indicating a potentially effective therapeutic option, either alone or as a sensitizer for other treatments.

## 1. Introduction

Uveal melanoma (UM) is a rare but an aggressive tumour of the eye that annually affects 5–7 adults per million in Caucasian populations, with mortality rates of approximately 40–50% within ten years of diagnosis [1,2,3]. The primary UM are effectively treated by surgery or radiotherapy. However metastatic UM is highly resistant to chemotherapeutic agents, limiting clinical treatment strategies [4]. Although the incidence of metastases at presentation is low, they subsequently develop with a peak incidence approximately three to five years after treatment of the primary tumour, underlining the importance of developing new and effective treatments to target this disseminated disease [5,6,7].

Non-random aberrations of chromosomes 1, 3, 6 and 8 are classically found in UM and are commonly used as reliable prognostic indicators [8,9,10], but these are mainly associated with the progression rather than initiation of UM [11,12]. In contrast, mutations in guanine nucleotide binding proteins *GNAQ* and *GNA11*, although not prognostic, occur in approximately 80–90% of UM and are considered an early, if not initiating event in UM [13,14,15,16]. These mutations produce constitutive activation of the ERK 1/2 / MEK and Yes Associated Protein (YAP) [17]. In cutaneous melanoma (CM), the targeted MEK inhibitor therapies are effective but, surprisingly they have shown little value in UM [18]. In addition, UM are resistant to other chemotherapy agents successful in the treatment of CM, notably the DNA inter-strand cross-linking (ICL) agent mitomycin-C (MMC). MMC prevents DNA replication by forming cross-links in DNA and is valuable as a treatment for a range of solid tumours, including conjunctival melanoma [19,20]. The single agent use of MMC is not however effective for most UM, possibly as previously shown, due to the deficient cytochrome-P450-mediated drug metabolism [21].

Therapies, such as ionising radiation (IR) and MMC, work by inducing DNA damage. A cell’s DNA damage response (DDR) involves a complex interplay of pathways designed to recognise and repair lesions occurring during normal cellular processes and those induced by agents, such as MMC. The double strand breaks (DSBs) arising either directly, or as a failure of single strand break repair, are the most lethal. The two main pathways for DSB repair are homologous recombination (HR) and non-homologous end joining (NHEJ). Repair by the latter is more error prone and consequently can lead to increased genetic instability. The selection of the pathway for repair depends on many factors, including the damaging agent, point in cell cycle and cell type. Furthermore, evidence suggests that a competitive relationship between the pathways can exist [22,23].

The authors have previously shown that low sister chromatid exchange (LSCE) frequency and elevated γH2AX foci formation are a feature of UM cell lines and primary short-term cultures (STCs), indicating that an irregularity in DSB repair exists in UM [24]. Furthermore, low RAD51 foci formation suggested low levels of endogenous HR in UM due to the low expression of Fanconi Anemia complementation group D2 (*FANCD2*) [25]. Given the relationship between HR and NHEJ during DSB repair, and the finding that *FANCD2* suppresses NHEJ [26], this study investigated whether UM have increased basal activity of the alternate DSB repair mechanism NHEJ, and whether its inhibition may represent a promising treatment option for UM.

## 2. Results

### 2.1. NHEJ is Elevated in UM

**Figure 1:**
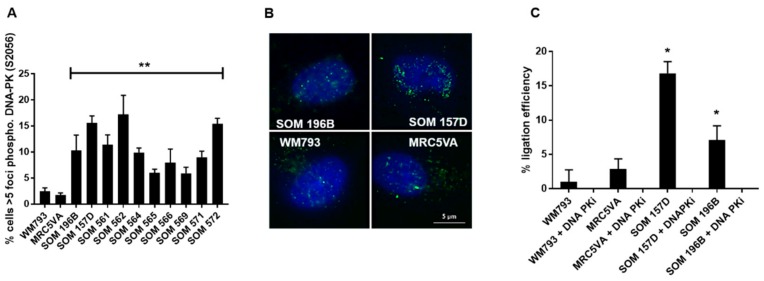
Non-homologous end joining (NHEJ) is elevated in uveal melanoma (UM) cell lines and short-term cultures (STCs). (**A**) UM cell lines and STCs showed greater formation of spontaneously activated serine 2056 DNA-PK foci compared to the control cells (**P < 0.01). The error bars represent the standard error from three independent trials. (**B**) Representative microscope images of cell lines stained for phosphorylated DNA-PKcs foci and counterstained with DAPI. The scale bar represents 5 microns. (**C**) NHEJ capacity assessed by the end-joining efficiency assay. If the plasmid had been re-ligated then colonies successfully formed. The end-joining efficiency (% ligation efficiency) was significantly elevated in UM cell lines compared with the control cell lines WM793 and MRC5VA (*P = <0.05). The end-joining capacity was eliminated in all cells treated with 10 µM NU7026 DNA-PK inhibitor, confirming the validity of the assay for measuring NHEJ-mediated ligation.

NHEJ in UM was first investigated by analysing the activation of DNA-PK measuring the foci formation of phosphorylated DNA-PKcs at the Serine 2056 (Ser2056) residue, an event that initiates NHEJ following DNA damage [27]. In this study, the spontaneous foci formation of Ser2056 DNA-PKcs was significantly higher in both UM cell lines and UM STCs when compared to the control cell lines, WM793 and MRC5VA (*P* < 0.01) (Figure 1). Furthermore, the ligation efficiency of NHEJ, estimated using a blunt-end cut plasmid transfection assay, demonstrated that DSB ligation efficiency was higher in both UM cell lines compared with the controls (Figure 1). Ligation efficiency in all cell lines was inhibited with 10 µM treatment of DNA-PK-inhibitor NU7026, confirming that the assay was effective in measuring end joining.

**Figure 2:**
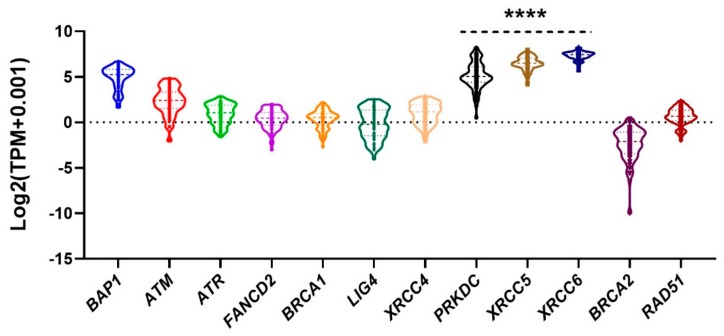
Confirmatory expression of NHEJ pathway genes in the Cancer Genome Atlas (TCGA). TCGA UM (*n* = 79) mRNA expression of genes involved in DNA repair pathways (*n* = 10). When compared to DSB-HR pathway genes *ATM, ATR, FANCD2, BRCA1, BRCA2* and *RAD51*, NHEJ pathway genes *PRKDC* (Alias *DNA-PKcs*), *XRCC5* and *XRCC6* were significantly highly expressed in UM (Tukey’s multiple comparisons test ****Adjusted *P* value < 0.0001). *BAP1* expression is normalized across both D3 and M3 UM, which accounts for the comparative upregulation.

The mRNA expression of genes associated with DNA repair pathways was analysed using the Cancer Genome Atlas (TCGA)-UVM RNAseq data (*n* = 79). For all UM, NHEJ pathway genes, DNA-PKcs (alias *PRKDC*), *XRCC5* and *XRCC6,* were significantly highly expressed compared to DSB-HR pathway genes *ATM, ATR, FANCD2, BRCA1, BRCA2* and *RAD51* (*P* < 0.0001) (Figure 2). It is, however, of interest that *LIG4* is not equally upregulated, and a recent study has also highlighted the upregulation of *PRKDC* but not *LIG4* [28].

The Kaplan–Meier survival plot was generated with respect to monosomy 3, a gain of 8q (M3G8q) and disomy 3 (D3) (Figure S1). As previously reported, M3G8q was associated with reduced survival (*P* <0.0001) and DNA-PK was found to be highly expressed in the cohort of M3G8q compared to D3 (*P* <0.0001) (Figure S1). Of note, the survival plots for the copy number deletion of *BAP1* (location 3p) and the amplification of *DNA-PK*/*PRKDC* (location 8q) matched exactly the plots for survival of M3G8q. The transcripts per million (TPM) value, however, for *DNA-PK*/*PRKDC* correlated to the copy number of 8q (Figure S2) offering an explanation for the association between the increased expression of *DNA-PK*/*PRKDC* with M3G8q. As the expression for all genes were normalized across both D3 and M3 UM. In combination with the experimental data (Figure 1), these findings demonstrate that NHEJ, from initiation through to completion, is upregulated in UM.

### 2.2. Inhibition of DNA-PK Mediated NHEJ is Toxic to UM and Induces Apoptosis

**Figure 3:**
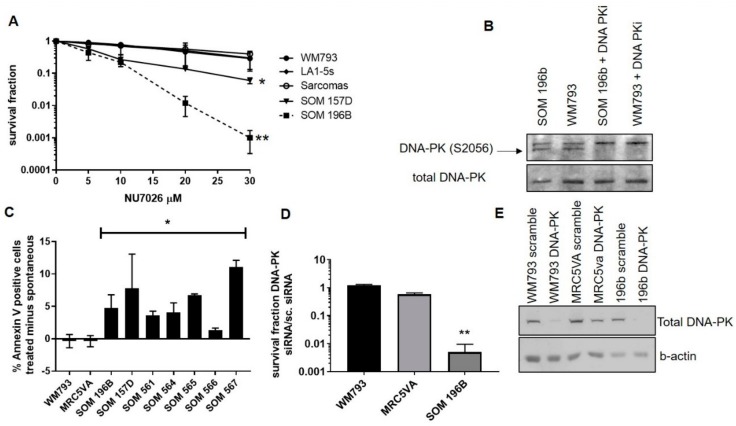
The sensitivity of UM cells to inhibition of DNA-PK. (**A**) UM cell lines SOM 157D and 196B were sensitive to DNA-PK inhibitor NU7026 when compared to the cutaneous melanoma (CM) cell line WM793, LA1-5s and an array of sarcoma cell lines (**P = <0.001 and *P =<0.05 respectively). (**B**) Inhibition of DNA-PK following treatment with NU7026 was confirmed by western blot in SOM 196B. (**C**) UM cell lines SOM 196B and SOM 157D and five available UM STCs show elevated apoptotic response in response to treatment compared to controls (*P = <0.05). (**D**) siRNA depletion of DNA-PK induced greater cytotoxicity in UM cell line SOM 196B compared with the control cell lines (**P = 0.003). Significance is measured against the scrambled siRNA control to account for the cytotoxic effects of transfection. Survival in the scrambled siRNA treated cultures was similar across all cell lines (*P* = >0.1). The error bars indicate the standard deviation in three independent trials. (**E**) DNA-PK inhibition by siRNA was confirmed by a western blot in the 3 cell lines tested, again using scrambled siRNA as the control.

All UM showed increased sensitivity to the DNA-PK inhibitor NU7026 when compared with the control cell lines WM793, melanocyte progenitor cell line LA1-5s and a series of sarcoma cell lines (A673, SKUT-1, SKLMS-1 and SW1353, grouped to simplify the data) (Figure 3). The 50% lethal dose (LD_50_) was 10 µM in UM cell lines SOM 157D and SOM 196B compared with 20–30 µM in controls (*P* = 0.04 and 0.02, respectively by a Student’s T-test). At 30 µM UM cell line 196B exhibited a 100-fold increased sensitivity versus the controls (*P* < 0.001 by a Student’s T-test). Due to the limited material available in UM STCs, these were assessed for sensitivity to NU7026 using the MTT assay. All ten UM STCs analysed were significantly more sensitive to NU7026 than the control (Figure S3) (*P* < 0.001 by a Student’s T-test). The inhibition of DNA PKcs phosphorylation following the treatment with 10μM NU7026 for 24 h was confirmed using a western blot analysis (Figure 3). Both UM cell lines were resistant to PARP inhibition in comparison to the HR deficient *BRCA1* mutant cell line COV362 (Figure S4).

As persistence of DSBs caused by impaired repair can trigger apoptosis, DNA-PK inhibition induced cell death, rather than just impeded growth, was confirmed by the Annexin V-FITC/FACS method. The Annexin V positive/PI negative cells in UM and the controls 48 h following the treatment with 10 µM NU7026 (the LD_50_ dose for UM), were significantly higher in the UM cell lines and STCs compared to the controls (Figure 3) (*P* < 0.05 by a Student’s T-test). As Annexin V positivity / PI negative is not always representative of early apoptosis, additional procedures (e.g., TUNEL) would provide further confirmation, but the higher levels in the UM cell lines following inhibition is suggestive of increased apoptosis. 

### 2.3. siRNA-Mediated Depletion of DNA-PKcs is Toxic to UM

Although NU7026 is highly selective against DNA-PK over other phosphatidyl inositol kinases (PIKs), it was necessary to determine whether the toxicity was due to specific targeting of DNA-PK or another unknown interaction. Therefore, the siRNA-mediated depletion of DNA-PKcs was performed. A control treatment of non-targeted scrambled siRNA (s.c.) accounted for the cytotoxic effects of transfection. The depletion of DNA-PKcs was highly cytotoxic to the UM cell line, SOM 196B, compared to both control cell lines, WM793 and MRC5VA (*P* = 0.003 by a Student’s T-test; Figure 3). The siRNA-mediated depletion of DNAPKcs was confirmed using a western blot analysis (Figure 3). The findings for siRNA were similar to those with the chemical inhibitor NU7026, confirming toxicity was indeed specific to the disruption of DNA-PK. 

### 2.4. Confirmation of UM Sensitivity to DNA-PK Inhibition with the Alternative Inhibitor NU7441

**Figure 4:**
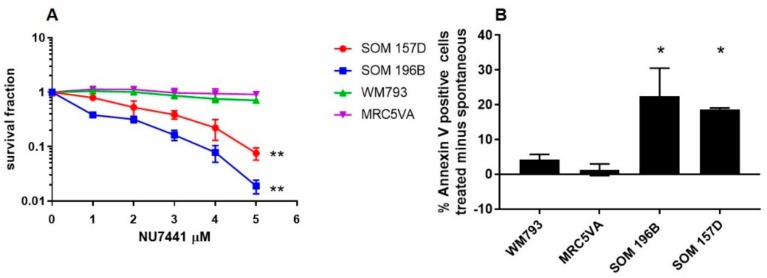
The sensitivity of the UM cell lines to DNA-PK inhibitor NU7441. (**A**) The UM cell lines SOM 157D and SOM 196B were sensitive to treatment with NU7441 DNA-PK inhibitor compared to the control cell lines (** *P* = 0.0088 and **P = 0.0043 respectively by a Student’s T test). (**B**) The percentage of apoptotic cells as suggested by Annexin V positivity was significantly increased in the UM cell lines SOM 157D and 196B in response to treatment with 2.5 µM NU7441 for 48 h compared to controls (* *P* = <0.05). The error bars indicate the standard deviation in three independent trials.

To support DNA-PK inhibition as a therapeutic option in UM, toxicity assays were performed using an alternative DNA-PK inhibitor, NU7441 (Figure 4). In pharmacokinetic studies in vivo, this compound has been shown to maintain clinically relevant concentrations in tumours significantly longer in comparison to NU7026 [29]. The UM cell lines SOM 157D and 196B were significantly more sensitive to treatment with NU7441 compared to the MRC5VA cells and WM793 cells (Figure 4). The LD50 values for SOM 157D and SOM 196B were 2.2µM and 0.8µM respectively, compared to > 5 µM in the control cells (*P* = 0.0088 and 0.0043 respectively by a Student’s T test). Additionally, the treatment with 2.5 µM NU7441 for 48 hours selectively induces apoptosis as suggested by the increase in Annexin V positive / PI negative cells in UM (Figure 4), as opposed to no increase in apoptosis in treated control cells MRC5VA and WM793 cells (*P* <0.05 by a Student’ T-test). 

### 2.5. DNA-PK Inhibition Potentiates the Cytotoxic Effects of IR in UM Cells and Sensitizes UM to ICL Agents

DNA-PK inhibitors have been shown to be effective in adjuvant therapy strategies in other tumours [30], and the findings of this study indicate a similar potential for UM. The DNA-PK inhibitor, NU7026 augmented the cytotoxic effects of IR in both the UM and control cell lines (Figure S5). At low doses of IR (2Gy) however, SOM 196 was sensitized three to four-fold in comparison to the control cell line WM793 (*P* = 0.002), while at higher doses of IR, there was no significant difference between their response, indicating that UM may respond more efficiently to adjuvant therapy strategies using DNA-PK inhibitors.

The potential for DNA-PK inhibitors in combined therapy also extends to previously ineffective therapies, including MMC, and in this study, the DNA-PK inhibitor NU7026 also sensitized the UM cell line SOM 196B to MMC (*P* = 0.02 at 20nM by a Student’s T-test). In comparison, any further cytotoxicity in the control cell line WM793 was not significant (*P* = 0.65) (Figure S5). In addition, most primary UM STCs were also found to be more resistant to MMC compared with the cell line WM793 (P<0.05 at 50nM), and most were sensitized to MMC with the co-administration of 10 µM NU7026 (*P* < 0.05 at 25 nM) (Figure S5). These effects were not restricted to MMC. Cisplatin, another DNA cross-linking agent, is not cytotoxic in UM, but the co-administration with 10 µM NU7026 increased its cytotoxic effects in the UM cell line, SOM 196B (*P* = 0.04 at 2 µM) (data not shown). Given the comparable response to cisplatin in the cell line and due to the limited cell numbers obtainable from STC of UM, the inclusion of UM STCs were restricted to the MMC investigation.

### 2.6. HR Functionality in UM Following Inhibition of NHEJ

As UM clearly have upregulation of NHEJ, the question arises as to whether LSCE are due to HR being outcompeted by NHEJ. An increase in SCE compared to the spontaneous levels was observed following treatment with NU7026 (*P* < 0.05), and the relative increase in SCE levels was comparable to that for the similarly treated WM793 line (Table 1). This finding suggests that the HR pathway is intrinsically functional in UM, and supports the notion of competition between the HR and NHEJ pathways [23,26]. 

## 3. Discussion

Previously, we found that UM has atypically low levels of SCE compared to both other cancer cell lines and normal cells [24]. The formation of SCE is most commonly associated with the HR pathway [31]. It would therefore be reasonable to hypothesise that UM is defective in HR, but as UM is notoriously resistant to both radiation and DNA-damaging agents (which HR deficient tumours are usually highly sensitive to [32]), an upregulation of an alternative DSB repair pathway to HR seems more probable. The data presented here, demonstrating the upregulation in vitro of functional NHEJ in both UM cell lines and STCs, is supported by gene expression analysis of the TCGA showing a significant increase in NHEJ proteins in comparison to HR proteins in 79 UM patients (Figure 2). Furthermore, the present study showed that all UM were more sensitive to the inhibition of DNA-PK and that apoptosis was induced when compared with other normal and cancer cell lines (Figure 3; Figure 4). 

Although DNA-PK, and therefore perhaps NHEJ, is vital to the survival of UM they are seemingly not intrinsically incapable of HR. A functional HR pathway in UM is indicated by the resistance to PARP inhibition and the increase in the observed levels of SCE following the inhibition of DNA-PK (Table 1, Figure S2). Although still lower than the control WM793 cells, the relative magnitude for the increase of SCE in UM was comparable to that of the control line (an effective doubling of all lines) suggesting that in all instances following the inhibition of DNA-PK, HR was prompted.

UM are not alone in having LSCE, as a reduced SCE formation has also been reported in Fanconi anaemia (FA) pathway deficient cells compared to proficient cells [33]. It has previously been shown that UM is deficient in the *FANCD2* protein associated with the FA pathway [25], and as one of the suggested roles of *FANCD2* is to inhibit NHEJ [26,34], its depletion in UM could therefore account for both the observed LSCE and overexpression of NHEJ. *FANCD2* has also previously been shown to influence HR and our previous data in UM supports this by demonstrating a reduction in spontaneous *RAD51* foci [25]. It is important to note that reduced HR, due to increased NHEJ, may have different consequences to where there is a fundamental inability to carry out HR, as for example is seen in *BRCA* deficient cells, since heterozygous carriers of *BRCA2* germline mutations exhibit increased spontaneous SCE despite impaired HR [35]. 

If UM are dedicated to NHEJ and equally committed to functional DNA-PKcs, an interesting therapeutic window is opened, as the cells deficient in DNA-PKcs have been shown to display heightened sensitivity to DSB-inducing agents [36]. In reverse, the over-expression of DNA-PK has been associated with increased chemo- and radio-resistance [37,38], potentially explaining the well documented resistance of UM to such therapies. Certainly, this study found that compound NU7026 sensitized UM cells to IR, MMC and Cisplatin. For UM, plaque radiotherapy achieves comparable survival rates to those obtained by enucleation [1], but a more effective response at a lower dose (Figure S3) may increase the overall response rates as well as reducing morbidity. Our observation that DNA-PK inhibitors sensitized UM to MMC contribute a further mode of ICL resistance to our previous finding of poor drug metabolism via altered CYTP450 [21]. Whether these modes are independent or share a common mechanism requires further investigation and may reflect heterogeneity with individual UM.

A potential competitive relationship between HR and NHEJ is proposed for other cancers [22,23,26] and here, this study shows that it likely exists in UM. This relationship may explain why in clinical trials Melphalan has been shown to have some effectiveness against UM [4], as resistance to Melphalan requires a fully functional HR capacity and indeed *FANCD2* expression [39]. UM, thus having a reliance/preference for NHEJ and reduced *FANCD2* expression, initially respond to Melphalan treatment. The reasons why they relapse later is less clear and it will be important to determine whether preferential use of NHEJ continues later in the disease or whether residual HR activity is enough to allow resistance to Melphalan. The exact mechanism for preference of UM for NHEJ repair is not clear at this point. Recent evidence has begun to suggest a consensus on how UM repair DNA damage with studies showing as the authors have, that Olaporib is not efficient alone on UM [40] and that *PRKDC* is upregulated, with NU7026 being an effective regulator of UM proliferation [28]. There are many points to explore in future studies. The use of assays, such as the DR-GFP/EJ5 assay, are required to investigate rigorously the HR status, whilst the anomaly of reduced *LIG4* expression may shed light on the interplay between HR and NHEJ in UM. Further investigations will provide a better understanding of the conundrum of selection for preferential DSB repair by UM and may provide new answers for the treatment of this difficult to treat melanoma.

## 4. Materials and Methods

### 4.1. Cell Culture

Two UM cell lines (Sheffield Ocular Melanoma, SOM 157D and SOM 196B) and short-term cultures (STC) of UM (SOM 561-572) were established from primary posterior UM biopsies from patients receiving surgical treatment at the Royal Hallamshire Hospital, Sheffield, UK, as described previously [41]. The clinico-pathological details of the STCs are provided in Table S1. The informed consent from all patients was obtained with ethical approval (SSREC 94/247 and 09/H1008/141) and the project followed the tenets of the Declaration of Helsinki. The cell lines and STCs were maintained as previously described [41]. In addition, the controls used were the CM cell line WM793 (a gift from Dr. M Herlyn, Wistar Institute, Philadelphia, USA), sarcoma cell lines SKUT-1, SKLMS-1, SW1353 and A673, fibroblast cell line MRC5VA, melanocyte progenitor cell line LA1-5s and BRCA-deficient ovarian cancer cell line COV362 (all ATCC). The MRC5VA cell line was chosen as the normal comparison for most studies since the growth of LA1-5s was more variable in the standard medium used for all lines. The STC of UM, because of their nature and insufficient cell numbers, were used only where possible to support the findings on the UM cell lines.

### 4.2. DNA Repair Protein Foci Formation Analysis

To measure the initiation of NHEJ repair, the cells were analysed for foci formation of phosphorylated DNA-dependent protein kinase (DNA-PK) (S2056, Abcam; 1:500 dilution) as previously described [24]. The percentage of the cells exhibiting greater than 5 foci per cell was calculated.

### 4.3. End-Joining Efficiency Assay

To measure ligation efficiency of DSBs in the cell lines, 5 × 10^6^ exponentially growing cells were electroporated with 100 ng of agarose gel-purified whole or Sal-I cut pUC19 plasmid (New England Biolabs, Hertfordshire, UK) in separate flasks and incubated for 48 h. The DNA was extracted using the Qiagen (Sussex, UK) mini-prep plasmid kit according to the manufacturer’s instructions and measured on a nano-drop spectrophotometer. Further, 100 ng of extracted DNA from each test was then separately transfected into competent TOP-10 *E. coli* cultures (Qiagen) on ice followed by 20 s in a 42 °C water bath, then 30 min on ice. The cultures were grown in SOB bacterial medium for 1 h at 37 °C with shaking at 225 rpm, then plated at various densities onto SOB-agar plates with 100 µg/mL ampicillin for each test. The transfection efficiency of the *E. coli* culture was calculated from the number of colonies resulting from a control transfection of agarose gel-purified whole pUC19 (equations given below). The colony counts from each cell line test were then converted into μg DNA in the extract using this transfection efficiency according to Qiagen’s guidelines. The ligation efficiency for each cell line was calculated as the fraction of total DNA in the Sal-I cut plasmid extract versus the whole plasmid extract. Additionally, a small volume of untransfected *E. coli* cells was plated onto ampicillin-free agar plates to control for contamination. The equations for calculating transfection efficiency:

**Transformation efficiency per µg DNA of the TOP10 *E. coli*** = (number of colonies/µg DNA) x (total transformation volume/volume plated)

**µg DNA per mammalian cell line extract** = [(number of colonies) × (total transformation volume/volume plated)]/Transformation efficiency per µg DNA of the TOP10 *E. coli*

### 4.4. The Cancer Genome Atlas (TCGA) UM Omics Data Analysis for Upregulation of NHEJ

The primary clinical data of UM was downloaded from the TCGA database (http://gdac.broadinstitute.org/) as well as from two published supplementary datasets on 19 December 2018 [42,43]. The RNAseq (transcripts per million (TPM) values, log2(x+0.001) transformed) and the copy number (gene-level gistic2 thresholded) data of UM were downloaded from the UCSC Xena TCGA Pan-Cancer Atlas Hub on 4 January, 2019 [44]. The UM samples were classified into two cohorts: Monosomy 3 chromosome 8q gain (M3G8q, N = 47) and Disomy 3 (D3, N = 32). The Kaplan–Meier estimator was used to determine the relationship among the cytogenetic abnormalities and patient survival. The statistical significance of the survival plots was evaluated using the log-rank (Mantel-Cox) test. The violin plot was used to visualize the distributions of the gene level normalized counts, and the statistical significance of the violin plots was evaluated using the unpaired t-test or Tukey’s multiple comparisons test. The values of *P* < 0.05 were considered significant. 

### 4.5. Toxicity Assays

Clonogenic survival assays: For measurement of sensitivity to DNA damaging agents, clonogenic survival assays were performed as previously described [21]. The incremental concentrations of the DNA-PK inhibitors (NU7026 Calbiochem (MERCK) Feltham UK and NU7441 Selleckchem Houston USA, and Poly(ADP-ribose) polymerases (PARP) inhibitor (Olaparib Calbiochem) were used. The alkylating agent mitomycin C (MMC) (Sigma Aldrich, Poole, UK) and the crosslinking agent cisplatin (Sigma Aldrich) were also tested and in all instances, the lines were cultured for 14 days. For tests using drugs dissolved in DMSO, all plates were treated with equal volumes of DMSO to control for toxicity caused by the solvent. The exposure to IR was achieved using a CIS IBL 437 Cesium-137 irradiator. For sensitization studies, the cells were treated with inhibitors for 2 hours prior to the damaging agent. MTT survival assays: As the clonogenic assay require an optimum number of cells for visualisation of the colonies, the slower growth rate of most STCs of primary UM were less amenable to this assay. Therefore, STCs from the primary UM were assayed by MTT as previously described [21]. For clonogenic assays, the survival fractions were calculated as the proportion of the cell colonies formed in the treated plates compared to the untreated control plate, and the average survival fractions were obtained. For the MTT assay, absorbance was measured at 570nm and for both assays, the controls were the relevant blank, either media or DMSO in media, depending on the solubility of the drug.

### 4.6. Protein Expression by Western Blotting

The protein expression of phosphorylated DNA-PK catalytic subunit (DNA-PKcs) and the total DNA-PK, SDS-PAGE and western blotting were performed on exponentially growing cell lines as previously described [21] using antibodies to DNA-PK (rabbit anti-human, Santa Cruz Biotechnologies) and phosphorylated DNA-PKcs at Serine 2056 (rabbit anti-human; Abcam, Cambridge, UK), each diluted 1:1000 in 5% bovine serum albumin solution. 

### 4.7. Annexin V Apoptosis Assay

To measure the apoptotic response to DNA-PK inhibitors, 1 × 10^6^ cells were seeded into 10 cm culture dishes and exposed to either 10 µM DNA-PK inhibitor NU7026, 2.5μM NU7441 or DMSO as a control and incubated at 37 °C/5% CO_2_ for 48 h. The cells were harvested and incubated with the Annexin V-FITC Fluorescence Activated Cell Sorting (FACS) assay kit (BD Pharmingen, Oxford, UK) according to the manufacturer’s instructions and measured as previously described, gating for Annexin V positive and PI negative cells [24].

### 4.8. siRNA Transfection

The control non-targeted scrambled oligonucleotide and siRNA oligonucleotides designed to target DNA-PK were purchased from Eurofins MWG Operon (Ebersberg, Germany).

(Sense:[GAUCGCACCUUACUCUGUU]-RNA-[TT]-DNA;

Antisense: [AACAGAUAAGGUGCGAUC]-RNA-[TT]-DNA).

Further, 10,000 cells grown in 6-well plates overnight were transfected with 70 nM scrambled siRNA or 70 nM DNA-PK targeting siRNA using Dharmacon SiPort reagent (Dharmacon, Thermo Fisher Scientific, Loughborough, UK) according to the manufacturer’s instructions. The cells were cultured in a normal growth medium for 48 h before re-plating for the clonogenic toxicity assay. The depletion was confirmed by western blotting.

### 4.9. Sister Chromatid Exchange Analysis

Furthermore, 10,000 cells were seeded into 6-well plates and treated with 10 µM NU7026 for 48 h (or DMSO for control cells). Following 48 h of treatment, the visualisation of SCEs was performed as previously described [24]. Approximately 30 cells per treatment group were scored. 

### 4.10. Statistical Analysis

The Student’s t-test or Tukey’s multiple comparisons test were applied to the data at a statistical significance threshold of 0.05.

## 5. Conclusions

Although this is a preliminary study, and additional functional investigations are required to support the findings, UM appear to upregulate NHEJ repair and hence, have a reliance on DNA-PK. This reliance seems to be an integral facet of UM, as it was evident in all UM regardless of other genetic changes. DNA-PK inhibitors were thus found to be a universally effective treatment against all the UM tested and could sensitize UM to the effects of IR and both MMC and Cisplatin. These findings suggest that the DNA-PK inhibitors provide a promising new line of therapy, either as a stand-alone agent, or in combination, for what has been previously a highly resistant and difficult to treat malignancy. Further studies are however required to support our findings and to establish what the HR status of UM is, and why UM seemingly have this apparent reliance on NHEJ.

## Figures and Tables

**Table 1:** DNA-PK inhibition raises sister chromatid exchange (SCE) levels in UM in a proportional manner.

	Mean SCE/Cell	Median SCE/Cell	Range
WM793 + DMSO	13.69	13	9–19
WM793 + DNAPKi	29.27	24	14–54
SOM 196B + DMSO	4.25	4	1–9
SOM 196 + DNAPKi	6.78	6.25	3–18
SOM 157d + DMSO	5.97	6	2–13
SOM 157d + DNAPKi	9.97	8	4–26

SCE analysis of UM cell lines demonstrates an increase in SCEs observed following treatment with 10μM NU7026 in both UM cell lines, despite levels of SCE remaining significantly reduced compared to spontaneous SCE levels in WM793 cells (*P = <0.05). Approximately 30 cells scored per condition. Error bars indicate the standard deviation in three independent trials.

## Supplementary Materials

The following are available online at https://www.mdpi.com/2072-6694/11/9/1278/s1, Figure S1: Overall Survival (OS) and PRKDC (DNA-PK) gene expression difference between the cohorts of Monosomy 3 chromosome 8q gain (M3G8q, *n* = 47) and Disomy 3 (D3, *n*= 32) TCGA UM (*n* = 79), Figure S2: UM are insensitive to PARP inhibition, Figure S3: Sensitisation of UM to IR/MMC by inhibition of DNA-PK, Table S1: Clinico-pathological details of UM short-term cultures.
