# Supplementary figures and images for: Increased Non-Homologous End Joining Makes DNA-PK a Promising Target for Therapeutic Intervention in Uveal Melanoma
Source: Cancers (Basel). 2019 Aug 30;11(9):1278. doi: 10.3390/cancers11091278 (PMC6769470; doi:10.3390/cancers11091278)

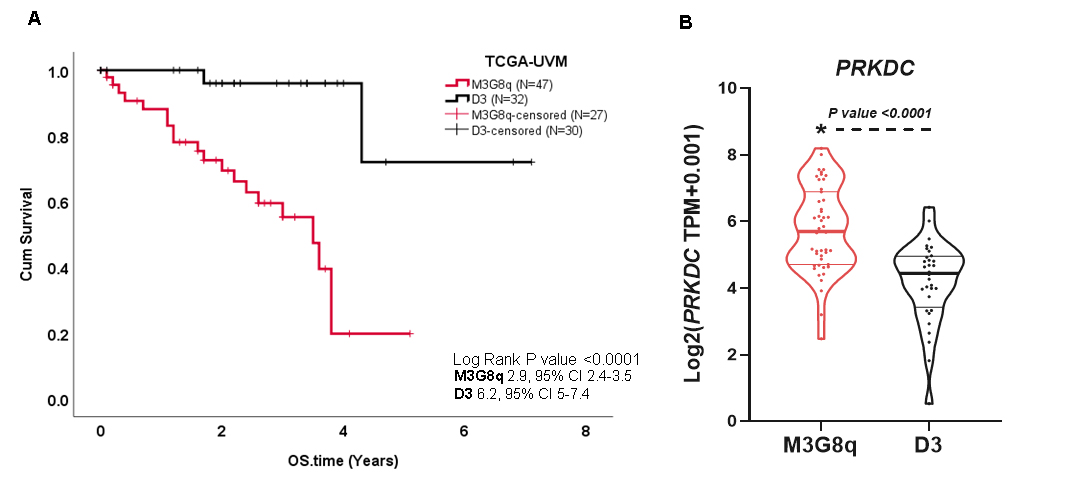

Supplement: Supplementary file 1 [file cancers-11-01278-s001.zip › supplemetary figures/Sup_Fig1.jpg]

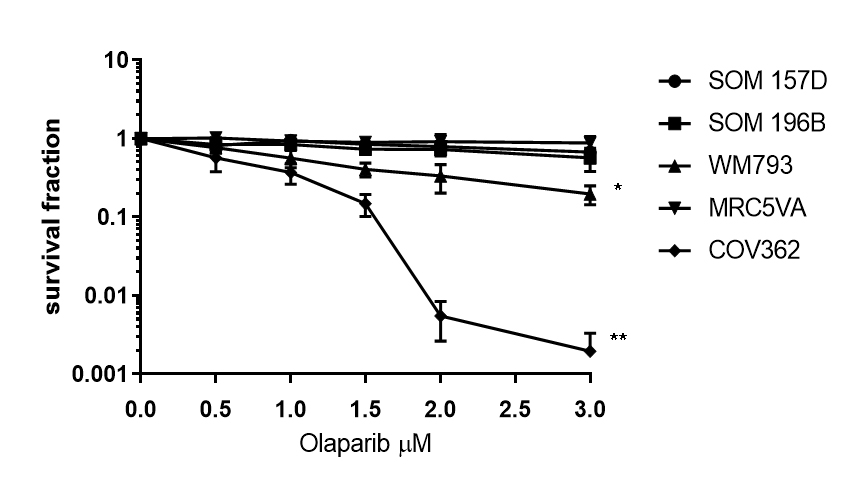

Supplement: Supplementary file 1 [file cancers-11-01278-s001.zip › supplemetary figures/Sup_Fig2.jpg]

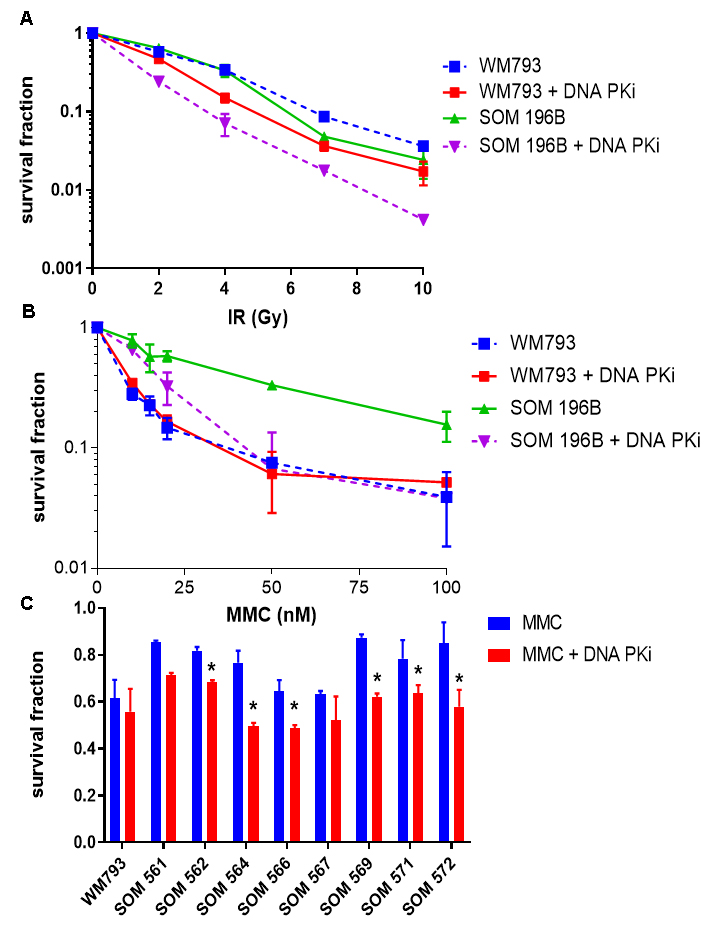

Supplement: Supplementary file 1 [file cancers-11-01278-s001.zip › supplemetary figures/Sup_Fig3.jpg]
